# Baseline Genomic Features in *BRAFV600*-Mutated Metastatic Melanoma Patients Treated with BRAF Inhibitor + MEK Inhibitor in Routine Care

**DOI:** 10.3390/cancers11081203

**Published:** 2019-08-18

**Authors:** Baptiste Louveau, Fanelie Jouenne, Coralie Reger de Moura, Aurelie Sadoux, Barouyr Baroudjian, Julie Delyon, Florian Herms, Adele De Masson, Laetitia Da Meda, Maxime Battistella, Nicolas Dumaz, Celeste Lebbe, Samia Mourah

**Affiliations:** 1 Pharmacogenomics Department, Assistance Publique-Hôpitaux de Paris (APHP), Saint Louis Hospital, 75010 Paris, France; 2 Université de Paris, INSERM UMRS 976, 75010 Paris, France; 3 Dermatology Department and Centre d’investigation clinique (CIC), Assistance Publique-Hôpitaux de Paris (APHP), Saint Louis Hospital, 75010 Paris, France; 4 Pathology Department, Assistance Publique-Hôpitaux de Paris (APHP), Saint Louis Hospital, 75010 Paris, France; 5 Université de Paris, INSERM UMRS 1165, 75010 Paris, France

**Keywords:** metastatic melanoma, BRAF inhibitor, MEK inhibitor, baseline genomic alterations

## Abstract

In *BRAF^V600mut^* metastatic melanoma, the combination of BRAF and MEK inhibitors (BRAFi, MEKi) has undergone multiple resistance mechanisms, limiting its clinical benefit and resulting in the need for response predicting biomarkers. Based on phase III clinical trial data, several studies have previously explored baseline genomic features associated with response to BRAFi + MEKi. Using a targeted approach that combines the examination of mRNA expression and DNA alterations in a subset of genes, we performed an analysis of baseline genomic alterations involved in MAPK inhibitors’ resistance in a real-life cohort of *BRAF^V600mut^* metastatic melanoma patients. Twenty-seven patients were included in this retrospective study, and tumor samples were analyzed when the BRAFi + MEKi therapy was initiated. The clinical characteristics of our cohort were consistent with previously published studies. The BRAFi + MEKi treatment was initiated in seven patients as a following-line treatment, and had a specific transcriptomic profile exhibiting 14 genes with lower mRNA expression. However, DNA alterations in *CCND1*, *RB1*, and *MET* were only observed in patients who received BRAFi + MEKi as the first-line treatment. Furthermore, *KIT* mRNA expression was significantly higher in patients showing clinical benefit from the combined therapy, emphasizing the tumor-suppressor role of *KIT* already described within the context of *BRAF*-mutant melanoma.

## 1. Introduction

*BRAFV600*-mutated (*BRAF^V600mut^*) lesions are currently described in approximately 50% of patients with metastatic melanoma, and are known to constitutively activate the MAPK (mitogen-activated protein kinase) pathway [1,2]. In the last few years, therapies targeting this pathway, such as those involving a BRAF inhibitor (BRAFi), have greatly improved the clinical outcome of *BRAF^V600mut^* metastatic melanoma [3,4,5,6,7,8]. However, the clinical benefit of these therapies has been limited by the emergence of multiple resistance mechanisms. Although the combination of a BRAFi with a MEK inhibitor (MEKi), which has become the standard of care [9], has improved outcomes, resistance has not been abolished.

Among the MAPK-targeting therapies’ resistance mechanisms described to date, reactivation of the MAPK pathway is the most frequent: it has been characterized in more than 70% of resistance cases in patients treated with BRAFi alone or with BRAFi + MEKi [10,11,12]. Frequent events leading to this reactivation suggest events such as *MAP2K1*, *MAP2K2*, or *NRAS* mutations, *BRAF* amplifications, or *NF1* inactivation. Other resistance mechanisms, such as PI3K/AKT pathway activation via *PTEN* and *AKT* mutations [13,14], *PDGFRB* and *IGFR1* overexpression [15,16], or production of HGF in the microenvironment [17], have also been described. Alterations in cell cycle genes, such as *CDKN2A*, have also been identified [14]. Moreover, targeted mRNA analyses have associated *BRAF* aberrant splice variants, overexpression of *MAP3K8*, and genes encoding tyrosine kinase receptors (RTKs), to resistance [18,19,20,21]. Recent studies using whole-exome sequencing highlight the multiplicity of resistance mechanisms within the same tumor, and a recent targeted approach of gene alterations uncovered new potential mechanisms of resistance [11,12,22,23]. However, data regarding resistance mechanisms in patients treated with MAPK-targeted therapies should be reinforced.

Considering these multiple and complex resistance phenomena and the rapid development of various therapies (targeted therapy, immunotherapy, etc.), the identification of baseline biomarkers and their association with patient clinical characteristics is crucial for identifying those who are more likely to benefit from these therapies. Using data from the BRIM and coBRIM studies, Wongchenko et al. [24] and Yan et al. [25] have recently focused on predictive genomic features, and revealed an association between an improved clinical response under BRAFi + MEKi treatment and higher levels of baseline immune response-related genes carrying *NF1* alterations. A MAPK pathway activity score predictive of clinical response to vemurafenib has also been proposed [26]. Regardless, despite the high heterogeneity in genomic profiles, these studies based on clinical trial data focus on the clinical course and do not investigate the association between genomic features and clinical attributes, such as the targeted therapy line.

In this retrospective study of a real-life cohort of *BRAF^V600mut^* metastatic melanoma patients initiating BRAFi + MEKi treatment, we applied a targeted molecular approach to describe baseline genomic alterations involved in resistance to MAPK-targeted therapies (mRNA expression, copy number, and mutations) and analyzed the association between these features and patient clinical characteristics.

## 2. Results

### 2.1. Patient Characteristics

**Figure 1:**
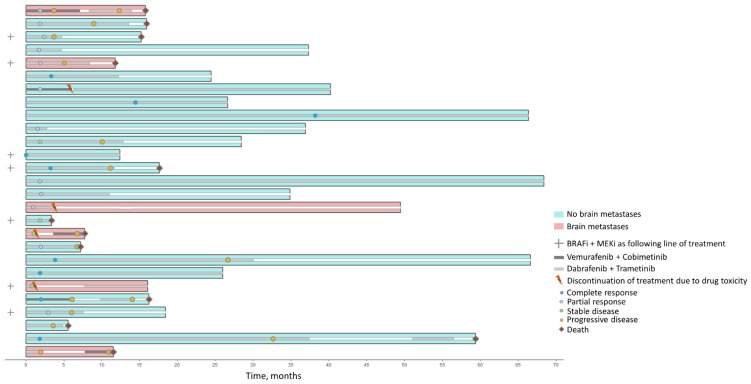
Clinical course of the 27 included patients. Events occurring during the BRAFi + MEKi treatment are shown. White bars indicate the absence of BRAFi + MEKi therapy. Patients were censored at the last available date of follow-up if death did not occur.

**Figure 2:**
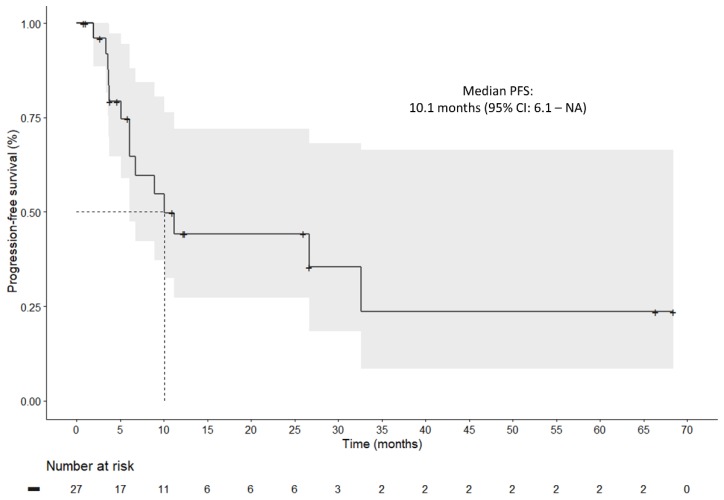
Kaplan–Meier survival plot for progression-free survival of the 27 included patients.

Twenty-seven (*n* = 27) *BRAF^V600mut^* metastatic melanoma patients were included in this study, and baseline tumor samples (prior to BRAFi + MEKi initiation) were collected. Patient characteristics are summarized in Table 1. Among the 27 patients, 24 (89%) presented with stage IV melanoma at initiation, comprising 16 (67%) M1c (American Joint Committee of Cancer 7th edition) cases. Brain metastases were observed for six (22%) patients and seven (29%) had elevated LDH levels. Twenty (74%) patients received BRAFi + MEKi as the first-line treatment, whereas seven (26%) received these targeted therapies as a following-line treatment. These seven patients all received immunotherapy prior to BRAFi + MEKi initiation. Compared to vemurafenib + cobimetinib (*n* = 3, 11%), dabrafenib + trametinib was mostly prescribed in our cohort (*n* = 24, 89%). Regarding safety, four (15%) patients experienced drug toxicity that led to treatment discontinuation. The clinical course, time under BRAFi + MEKi treatment, and best-observed response during follow-up are summarized in Figure 1. The median follow-up time in our cohort was 18.4 (lower limit = 3.4; upper limit = 68.4) months. The best observed response under therapy was nine cases of complete response (33%), 12 cases of partial response (44%), and four cases of stable disease (15%), with an overall response rate (ORR) of 78%. Median progression-free survival (PFS), defined as the time between BRAFi + MEKi initiation and the occurrence of disease progression or death (whichever occurred first) under treatment, was estimated at 10.1 months (95% CI: 6.1; NA) (Figure 2). Univariate analyses were performed to search for associations between PFS and clinical baseline characteristics. The presence of brain metastases (*p* < 0.01) and LDH levels (*p* < 0.01) at therapy initiation were significantly associated with PFS.

### 2.2. Baseline DNA Analysis (Mutations and CNVs)

**Figure 3:**
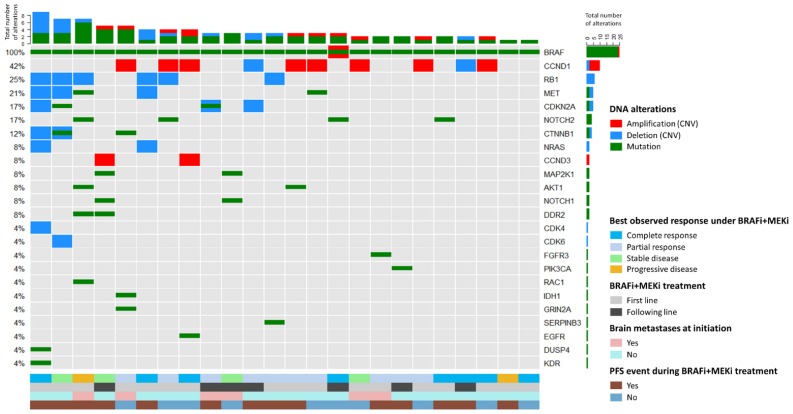
Landscape of baseline DNA alterations (mutations and copy number variations) for the 24 patients with available data. Amplifications and deletions were defined as CNV > 5 and CNV < 0.5, respectively. Patients are ranked according to the total number of alterations detected.

Next-generation sequencing (NGS) and copy number analyses targeting 78 genes involved in the MAPK, PI3K, RTK, and cell cycle pathways (Supplementary Table S1) were performed on baseline tumor samples from 24 patients (3 missing because of insufficient material). This analysis led to the detection of 82 alterations, including 51 (62%) mutations and 31 (38%) copy number variations (Figure 3). Copy number variations in *CCND1* were the most frequent DNA alteration observed, with both amplifications and deletions in eight and two of the 24 samples, respectively. *RB1* deletions were also highlighted in six (25%) samples. *NOTCH2* exhibited the highest mutation rate, with four (17%) mutations detected. Details regarding all baseline mutations detected by NGS (amino acid change, allele frequency) are provided in Supplementary Table S2.

Analyses were performed to search for a possible association between the number or type of alterations in our screened genes and clinical variables. Comparison of patients harboring a high number of DNA alterations (≥4) vs. those with a low number of alterations (≤3) revealed that patients in the group with ≥4 alterations (7/8, 88%) were more likely to have received BRAFi + MEKi as the first-line treatment. Moreover, *CCND1* amplifications, *RB1* deletions, and *MET* alterations were only retrieved for those patients treated with BRAFi + MEKi as a first-line. No association (type or total number of alterations) was observed for the presence of brain metastases, a high LDH level, best observed response, or occurrence of a PFS event under BRAFi + MEKi treatment. Patients with and without a PFS event presented a mean of 4 and 2.9 DNA alterations, respectively (*p* = 0.19). Similarly, responders and nonresponders harbored a mean of 3.3 and 4.3 alterations, respectively (*p* = 0.42). Analyzing only those without brain metastases confirmed this observation. In this subgroup, patients with and without a PFS event presented a mean of 4.0 and 2.7 alterations, respectively (*p* = 0.19). Furthermore, responders and nonresponders harbored a mean of 3.3 and 4.7 alterations, respectively (*p* = 0.57).

### 2.3. Baseline mRNA Expression Analysis

**Figure 4:**
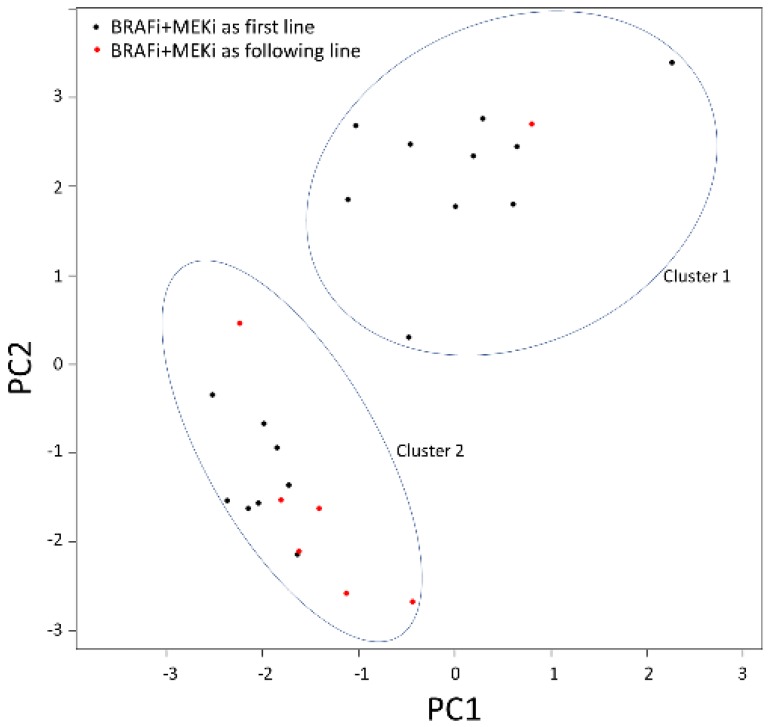
Principal component analysis of mRNA expression for the 25 patients with available data (∆Ct, normalization on *B2M*). Patients from Cluster 1 were more likely to have been treated with BRAFi + MEKi as a first-line treatment.

mRNA expression analysis was performed on 29 genes involved in the RAS–RAF–MAPK pathway, cell cycle, or apoptosis and implicated in MAPK inhibitor resistance mechanisms (Supplementary Table S1). Data were generated for baseline tumor samples from 25 patients (two patients with noninformative data). Principal component analysis (PCA) of the ∆Ct values was performed, revealing two clusters with different mRNA expression profiles (Figure 4).

**Figure 5:**
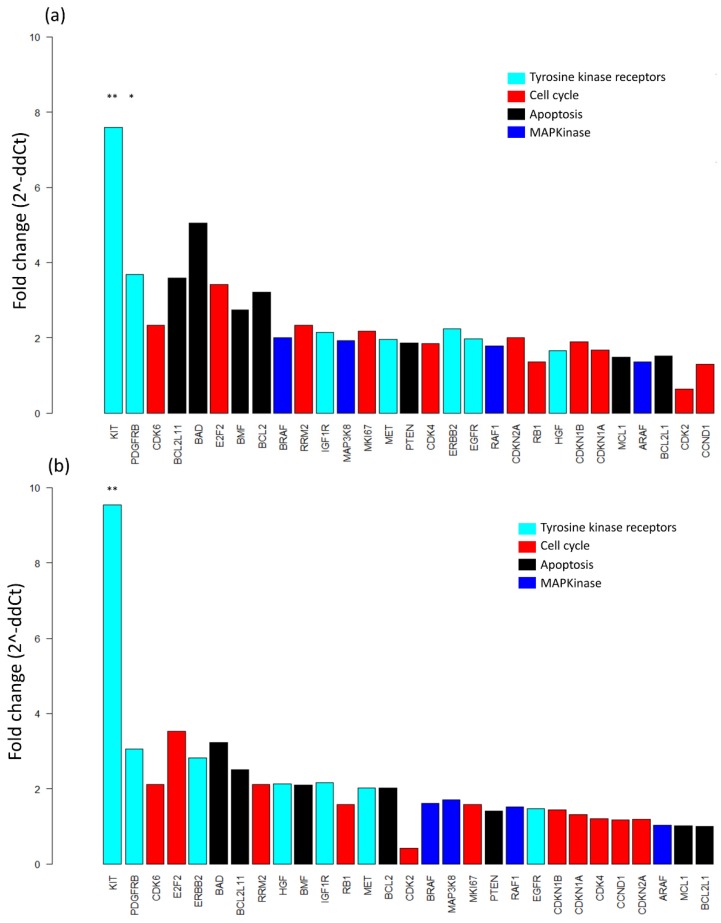
Differential gene expression analysis according to clinical course. (**a**) Differential gene expression in patients with no progression free survival (PFS) event vs. patients with PFS event (*n* = 25). (**b**) Differential gene expression in patients with no PFS event vs. PFS event (only patients with no brain metastases, *n* = 20). Differences in gene expression are expressed as fold change (2^-∆∆Ct), and genes are ranked by their level of significance. ** *p* < 0.01, * *p* < 0.05.

In addition, differential transcript expression analysis was performed to identify expression profiles associated with clinical characteristics and to detect differentially expressed genes. Briefly, mRNA expression fold changes (2^-∆∆Ct) between predefined subgroups were calculated to assess relative quantification. First, patients were compared according to their clinical course during the BRAFi + MEKi treatment: occurrence of a PFS event vs. no PFS event and clinical response (complete or partial response) vs. no clinical response (stable or progressive disease). For most tested genes, higher mRNA expression levels were observed in patients with no PFS event vs. PFS event, with two genes coding for RTKs, *KIT*, and *PDGFR*, being significantly overexpressed in those with no PFS event (*p* < 0.01; Figure 5a). This association remained significant for *KIT* after false discovery rate (FDR) correction. However, no significant differential gene expression was found when comparing patients with or without a clinical response. These observations were confirmed when considering only patients with no brain metastases (*n* = 20). In this subgroup, *KIT* expression was significantly higher in patients with no PFS event vs. PFS event (*p* < 0.01; Figure 5b), though with no significant difference according to the occurrence of a clinical response. Due to the small number of patients with available data (*n* = 5), differential analysis was not performed specifically for the subgroup of “brain metastases”.

**Figure 6:**
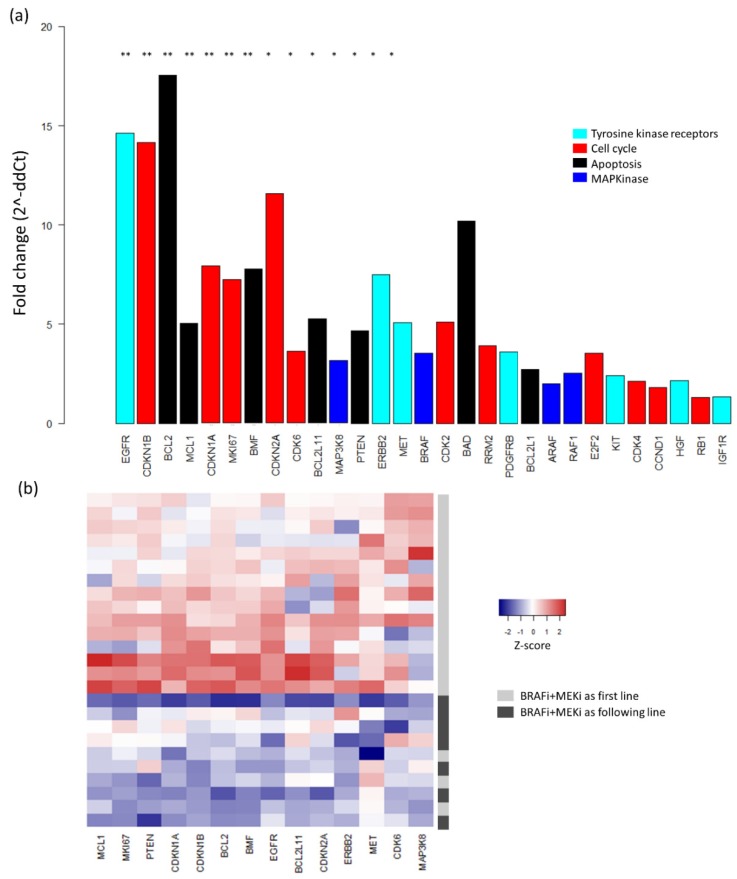
**Differential gene expression analysis according to line of treatment** (**a**) Differential gene expression in patients with BRAFi + MEKi as the first-line vs. patients treated with BRAFi + MEKi as the following-line of treatment. Differences in gene expression are expressed as fold change (2^-∆∆Ct), and genes are ranked by their level of significance. ** *p* < 0.01, * *p* < 0.05. (**b**) Heatmap of mRNA expression. Colors represent the relative expression of a gene in each sample centered on the mean and scaled to the standard deviation (red: high and blue: low).

Second, analysis was performed to identify differential gene expression according to baseline clinical characteristics. Interestingly, comparison of the two clusters described in PCA showed that patients from Cluster 1 were more likely to have been treated with BRAFi + MEKi as the first-line than the following-line (91% vs. 57%, *p* = 0.06; Figure 4). According to differential expression analysis, 14 genes showed significantly higher expression in patients receiving BRAFi + MEKi as the first-line treatment vs. patients with BRAFi + MEKi as the following-line treatment, with seven remaining significant after FDR correction (Figure 6a). Using these 14 genes, an unsupervised heatmap was then plotted, confirming these different mRNA expression profiles between patients treated with BRAFi + MEKi as the first- and following-line (Figure 6b). Nonetheless, statistical significance for differential expression in patients with brain metastases vs. no brain metastases, or in patients with high LDH levels vs. low LDH levels, was not observed for any gene.

## 3. Discussion

Studies on the predictive potential of baseline genomic features in metastatic melanoma patients treated with targeted therapies have thus far primarily been performed using phase III clinical trial data. As a complement, data from patients followed as part of routine care are needed to enhance our understanding of these genomic features and their association with clinical characteristics.

Here, we present an exploratory analysis of a cohort of *BRAF^V600mut^* metastatic melanoma patients initiating BRAFi + MEKi treatment followed in a real-world setting. A median PFS of 10.1 months and a high rate of clinical response under BRAFi + MEKi (21/27 patients, 78%) were observed in our cohort, which is consistent with previous phase III trials [7,8,27,28] and recently published real-world studies [29,30,31]. Regarding clinical baseline characteristics, 16 patients (59%) were at stage IVM1c upon combined therapy initiation, which is similar to the rate reported by Long et al. [8] in the COMBI-D trial or by Luke et al. [31] in a real-world setting. The number of patients with brain metastases or with high LDH levels was also consistent with previous studies. Moreover, unlike clinical trials, seven patients (26%) who underwent previous systemic cancer therapy were included in our study, allowing a better representation of a real-life population. Indeed, in routine care, patients are likely to have undergone multiple treatments prior to BRAFi + MEKi [30].

Combining DNA mutations, copy number, and mRNA expression analyses, we conducted a targeted genomic approach to examine tumor samples prior to BRAFi + MEKi initiation. The studied genes were chosen based on the literature and were selected according to their potential implication in resistance to the MAPK inhibitor [32,33,34,35]. *NRAS* and *MAP2K1* alterations concomitant with *BRAF* mutations were found in four patients (15%), which is consistent with the 24% rate reported by Johnson et al. in relapsed tumors [12]. However, we did not observe any associations between the type or total number of DNA alterations among our screened genes and clinical course under treatment. Although these observations must be considered carefully due to the small size of our cohort, they are consistent with recent analyses of vemurafenib + cobimetinib-treated patients from the BRIM-2, BRIM-3, BRIM-7, and coBRIM studies. Indeed, tumor mutational load, in addition to *BRAF* and *CDKN2A* alterations are reportedly similar between patients with complete response and those with rapid progressive disease [25]. Regarding mRNA expression, studies have evaluated levels in relapsed tumors, but few have characterized levels prior to MAPKi treatment. Overall, such predictive signatures are crucial for the identification of patients that are more likely to benefit from targeted therapy. Using data from the BRIM and coBRIM studies, Wongchenko et al. [24] and Wagle et al. [26] recently proposed an immune expression signature and a baseline MAPK pathway activity score, respectively, as predictors of clinical course under vemurafenib treatment. Our targeted approach has previously associated mRNA expression levels of a subset of genes (including RTKs, *PGFRB*, *EGFR*, and *ERBB2*) with survival in a real-life cohort of BRAFi-treated metastatic melanoma patients, highlighting a tumor proliferation/metabolic rate that might render it more sensitive to BRAFi [23]. In the present study, using the same targeted approach and focusing on patients treated with BRAFi + MEKi, we highlight the same trend with genes presenting higher expression in patients achieving clinical benefit from BRAFi + MEKi (no PFS event). Significance was observed for only a few genes, which may be related to the small size of our cohort and the occurrence of treatment discontinuation (adverse events or medical decision). Nonetheless, an interesting finding from our study is that one RTK-related gene, *KIT,* showed a high level of statistical significance. Low levels of *KIT* expression have been widely described in most cases of cutaneous melanoma, especially in *BRAF^V600mut^* melanoma, due to the endogenous activation of MAPK activation by *BRAF* mutations [35,36,37]. Furthermore, the loss of *KIT* has recently been described as a mechanism leading to increased *BRAF^V600E^* signaling. In *BRAF^V600E^* cells, *KIT* suppresses the RAS/MAPK pathway activity mediated by *BRAF* activation, and acts as a tumor suppressor [38]. As the majority of resistance mechanisms to BRAFi + MEKi are related to the MAPK pathway [11,22], we may assume that this baseline tumor-suppressor effect of *KIT* is emphasized in our cohort, improving the clinical course under BRAFi + MEKi. This alternative activation of the RAS/MAPK pathway via activated RTKs within the context of *BRAF* mutants has also been described by Grimm et al. [39].

We further analyzed the associations between genomic alterations and clinical baseline characteristics. Interestingly, *CCND1* amplifications, *RB1* deletions, and *MET* alterations were only observed for patients receiving BRAFi + MEKi as a first-line therapy, and none of the patients initiating BRAFi + MEKi as the following-line harbored these alterations. All these latter patients received and discontinued their immunotherapy treatment prior to BRAFi + MEKi initiation, suggesting that resistance mechanisms to immunotherapies may select tumor cells devoid of such genetic alterations.

Moreover, a difference in the baseline mRNA expression profile between patients treated with BRAFi + MEKi as the first-line and the following-line was also highlighted. Genomic biomarker studies performed to date in the field of melanoma have focused on first-line-treated patients, whereas little is known about tumor genomics in pretreated patients. In our study, we show that several genes involved in the cell cycle (*CDKN1B*, *CDKN1A*, *MKI67*) and apoptosis (*BCL2*, *MCL1*, *BMF*) pathways were more highly expressed in patients treated with BRAFi + MEKi as a first-line. Consistent with previous results [40], and because patients undergoing BRAFi + MEKi as the following-line do not present a satisfying response to previous treatment, our finding reveals the importance of presenting a high mRNA expression profile to obtain a benefit from immunotherapy. More broadly, these data show the importance of considering the treatment history when studying predictive genomic biomarkers and resistance mechanisms to targeted therapy.

Our targeted approach, combining DNA and mRNA alterations analyses, revealed the potential role of *KIT* as a predictive biomarker of response to BRAFi + MEKi. Within the context of metastatic melanoma, with frequent treatment discontinuation, our data also highlight the importance of performing tumor genomic analysis when switching therapy to account for molecular changes during successive targeted therapies/immunotherapies. However, considering the small size of our cohort, further studies are needed to support our findings.

## 4. Materials and Methods

### 4.1. Patients and Samples

Twenty-seven stage III or IV *BRAF^V600mut^* metastatic melanoma patients initiating combined treatment of BRAFi + MEKi as first- or following-line therapy and followed at Saint Louis Hospital (Paris, France) were included in this retrospective study. All patients were enrolled in MelBase, a multicenter French clinical database with a biobank dedicated to the prospective follow-up of advanced melanoma patients since March 2013. MelBase was approved by the French ethics committee (CPP Ile-de-france XI, n°12027, 2012) and registered in the NIH clinical trials database (NCT02828202). Written informed consent was obtained from all patients for their participation, including collection and analysis of their data. Patients were followed by a dermato-oncologist as part of the routine care at the Oncodermatology unit, and clinical response was evaluated using response evaluation criteria in solid tumor (RECIST) criteria [41]. For each patient, baseline (prior to BRAFi + MEKi initiation) tumor tissues were collected, stored as formalin-fixed, paraffin-embedded (FFPE), or frozen samples, and processed as previously described [23]. Samples harboring below 50% of tumor cells were macrodissected.

### 4.2. DNA and mRNA Extractions

DNA and mRNA were extracted from tumor samples as previously described [23]. Frozen tumor samples were processed with Maxwell RSC Tissue DNA and Maxwell RSC simplyRNA Tissue kits according to the manufacturer’s protocol for DNA and mRNA extraction, respectively (Promega, San Luis Obispo, CA, USA). A NanoDrop ND-1000 spectrophotometer (NanoDrop Technologies, Wilmington, NC, USA) was used for DNA and mRNA quantification and qualification. A Qubit 2.0 fluorometer (Thermo Fisher Scientific, Waltham, MA, USA) was also used for DNA quantification. 

### 4.3. Copy Number Variation and Transcript Analyses

An off-the-shelf commercial personalized Human quantitative PCR (qPCR) SignArrays^®^ 96 system (qPCR SignArrays^®^96 VPR1H1 kit, Anygenes, Paris, France) was used for gene copy number variation (CNV) and mRNA expression analyses. The PCR mixture, including 10 μL of Perfect MasterMix SYBR Green^®^, 8 μL of PCR grade water, and 2 μL of DNA (or cDNA after reverse transcription), was dropped into each well of the qPCR array. Amplification was performed using a LightCycler 480 (Roche, France) in duplicate according to the following sequence: (1) 10 min at 95 °C; (2) 40 cycles of 10 s at 95 °C; and (3) 30 s at 60 °C. The studied genes, involved in the RAS–RAF–MAPK pathway, cell cycle, or apoptosis as previously described [23], were selected for their validated or suggested role in BRAF inhibitor resistance. mRNA expression and copy number analyses were performed on 29 and 12 genes, respectively (Supplementary Table S1).

First-strand complementary DNA (cDNA) was synthesized from 1 µg of total RNA using a High-Capacity cDNA Archive Kit (Applied-Biosystems, Life Technologies, Carlsbad, CA, USA) according to the manufacturer’s protocol. The obtained cDNA samples were diluted 6 times for the qPCR assay. The raw mRNA expression data were normalized according to the ΔCt method (Ct: threshold cycle of amplification), as implemented in the ‘HTqPCR’ Bioconductor package (v1.36.0, https://www.bioconductor.org/packages/release/bioc/html/HTqPCR.html) [42]. For each targeted gene for each sample, mRNA expression values were generated in duplicate, and *beta-2-microglobulin (B2M)* was used as a housekeeping gene for normalization.

Gene copy number was quantified by comparison to *glyceraldehyde 3-phosphate dehydrogenase* (*GAPDH*) as an internal control. Two sets of primers were used for each gene, and relative copy numbers were calculated using the ΔΔCt method. Differences in the Ct of the targeted gene and *GAPDH* were assessed for each sample and compared to those in a reference pool of normal genomic DNA obtained from 10 samples of benign tissue. Relative copy number was calculated following the 2^-ΔΔCt formula, and conversion to absolute copy numbers was performed by assigning a value of 2 (diploid) to the reference pool and multiplying the relative copy number of samples by a factor of 2. Thresholds of 5 and 0.5 were set to define DNA amplification and DNA deletion, respectively [43,44].

### 4.4. Next-Generation Sequencing (NGS)

Targeted sequencing was performed with a customized AmpliSeq™ NGS panel (Thermo Fisher Scientific) designed with the Ion AmpliSeq™ designer software (Life Technologies). Seventy-four genes involved in the MAPK, PI3K, RTK, and cell cycle pathways were targeted (Supplementary Table S1). This panel of 35 kb generates 328 amplicons with an average size of 120 bp and a mean coverage of 92.79%. Sequencing amplicon libraries were synthesized from 50 ng of genomic DNA isolated from tissue samples using an Ion AmpliSeq™ Library Kit 2.0 (Thermo Fisher Scientific) and indexed with an Ion Xpress Barcode Adapters Kit (Life Technologies) following the manufacturer’s instructions. Amplification quality was defined with an Agilent 2100 BioAnalyzer. The library amplicon pool was sequenced with the Ion PGM™ Sequencer (Thermo Fisher Scientific) using Ion PGM Hi-Q Chef chemistry (single-end reads of 120 bp) and a 318-V2-BC sequencing flow cell. Base calling, alignment to the hg19 human reference sequence and variant calling were performed using Torrent Suite Software v5.8.0 (Thermo Fisher Scientific). ANNOVAR was employed for annotation through the Galaxy-APHP interface. Integrative Genomics Viewer (IGV) was used to visualize the read alignment and confirm the variant calls. Variant selection was performed using Alamut (Interactive Bio-Software) with the following criteria: (1) location in exonic or splice region; (2) nonsynonymous coding variant; (3) no previous annotation in the 1000 genomes project, ESP and ExAC databases or with an allelic frequency < 1%; (4) coverage ≥ 500×; (5) variant allele fraction (VAF) ≥ 5%; and (6) strand bias < 95%.

### 4.5. Statistical Analysis

Clinical characteristics are described in terms of median [IQR] for quantitative variables and in terms of number (%) for qualitative variables. PFS was defined as the time between BRAFi + MEKi initiation and disease progression or death of any cause under treatment, whichever occurred first. Patients were censored at the time of BRAFi + MEKi interruption. PFS was estimated using the Kaplan–Meier method, and comparisons were performed with the log rank test. Copy number variations were dichotomized as amplifications (>5) and as deletions (<0.5). The number of DNA alterations between groups was compared using a *t*-test. Differential expression analysis between groups of interest was performed on normalized data using ‘HTqPCR’ Biopackage [42]. ΔΔCt, defined as ΔCt_group1_−ΔCt_group2_, was calculated for each targeted gene, and the fold change between groups was set as 2^-ΔΔCt. A *t*-test was performed to assess any difference between groups, and FDR was used for multiple test corrections. Statistical significance was set at *p* ≤ 0.05, and all analyses were performed with R 3.5.1 (R foundation for statistical computing, Vienna, Austria).

## 5. Conclusions

Despite the small number of patients included in our study, our targeted genomic approach in a real-life cohort of patients initiating the BRAFi + MEKi treatment revealed a differential baseline transcriptomic profile with higher mRNA expression in *BRAF^V600mut^* metastatic melanoma patients receiving this combined therapy as a first-line treatment. *KIT* was found to be overexpressed in patients with an improved clinical course, suggesting its potential predictive value. Further studies on larger cohorts are needed to support our findings.

## Figures and Tables

**Table 1:** Patient characteristics at BRAFi + MEKi initiation.

	All Included Patients
	(*n* = 27)
**Male sex**	13 (48%)
**Age at melanoma diagnosis, years**	49.3 (35.5; 58.8)
**Melanoma subtype**	
Superficial spreading melanoma	15 (56%)
Nodular melanoma	5 (19%)
Others	2 (7%)
Undetermined	5 (19%)
**Breslow thickness, mm**	3 (1.4; 5.2)
**Ulceration**	11/24 (46%)
**Age at initiation, years**	49.9 (40.8; 62.7)
**Disease stage**	
Unresectable stage III	3 (11%)
Unresectable stage IV	24 (89%)
M1c	16 (67%)
**Brain metastases**	6 (22%)
**Elevated lactate dehydrogenase level**	7/24 (29%)
**Number of disease sites**	
≤2	13/24 (54%)
≥3	11/24 (46%)
**BRAFi + MEKi combination initiated**	
Dabrafenib + trametinib	24 (89%)
Vemurafenib + cobimetinib	3 (11%)
**BRAFi + MEKi as first-line treatment**	
First-line treatment	20 (74%)
Following-line treatment	7 (26%)
**Previous line of treatment (if any)**	
BRAFi monotherapy	3 (11%)
Anti-CTLA4	2 (7%)
Anti-PD1/PDL1	6 (22%)
Anti-CTLA4 + anti-PD1	2 (7%)
Others	1 (4%)

Data are median (interquartile range—IQR) and number (%). Elevated lactate dehydrogenase levels are defined as >480 UI. BRAFi: BRAF inhibitor, MEKi: MEK inhibitor, and mm: millimeters.

## Supplementary Materials

The following are available online at https://www.mdpi.com/2072-6694/11/8/1203/s1, Table S1: List of studied genes, Table S2: Mutations detected by next-generation sequencing (panel of 74 genes) on the 24 baseline samples.
