# Supplementary material for: Baseline Genomic Features in BRAFV600-Mutated Metastatic Melanoma Patients Treated with BRAF Inhibitor + MEK Inhibitor in Routine Care
Source: Cancers (Basel). 2019 Aug 18;11(8):1203. doi: 10.3390/cancers11081203 (PMC6721518; doi:10.3390/cancers11081203)
Supplement: Supplementary file 1 [file cancers-11-01203-s001.pdf]

Supplemental Materials

Baseline Genomic Features in *BRAFV600*-Mutated Metastatic Melanoma Patients Treated with BRAF Inhibitor + MEK Inhibitor in Routine Care

Baptiste Louveau, Fanelie Jouenne, Coralie Reger de Moura, Aurelie Sadoux, Barouyr Baroudjian, Julie Delyon, Florian Herms, Adele De Masson, Laetitia Da Meda, Maxime Battistella, Nicolas Dumaz, Celeste Lebbe and Samia Mourah

Table S1. List of studied genes.

Mutation analysis (Next Generation Sequencing)			DNA Copy number analysis	mRNA expression analysis
ABL1	*GNAQ*	*RAC1*	*BRAF*	*BRAF*
AKT1	*GRIN2A*	*RAF1*	*NRAS*	*RAF1*
AKT2	*GRM3*	*RASA2*	*MAP2K1*	*ARAF*
AKT3	*HOXD8*	*RET*	*MET*	*PDGFRB*
ALK	*HRAS*	*RPS27*	*CDKN2A*	*IGF1R*
ARAF	*IDH1*	*SERPINB3*	*CDK4*	*MET*
BRAF	*IGF1R*	*SNX31*	*CDK6*	*HGF*
BTK	*JAK2*	*STAT3*	*CCND1*	*KIT*
CCND1	*KDR*	*STK11*	*CCND2*	*EGFR*
CDK4	*KIT*	*STK19*	*CCND3*	*ERBB2*
CDKN2A	*KRAS*	*TACC1*	*RB1*	*MAP3K8*
CD274	*MAP2K1*	*TERT*	*CTNNB1*	*MKI67*
CTNNB1	*MAP2K2*	*TRRAP*		*E2F2*
CXCR4	*MDM2*	*WT1*		*RB1*
DDR1	*MET*			*CDK2*
DDR2	*MITF*			*CDK4*
DDX3X	*MRPS31*			*CDK6*
DUSP4	*NOTCH1*			*CCND1*
EGFR	*NOTCH2*			*RRM2*
ERBB2	*NRAS*			*BCL2*
ERBB3	*PDGFRA*			*BCL2L1*
ERBB4	*PDGFRB*			*BCL2L11*
EZH2	*PHLPP1*			*BMF*
FBXW7	*PIK3CA*			*MCL1*
FERMT3	*PIK3CG*			*BAD*
FGFR1	*PIK3R1*			*PTEN*
FGFR2	*PIK3R2*			*CDKN1A*
FGFR3	*PLCG2*			*CDKN1B*
FLT3	*PPP6C*			*CDKN2A*
GNA11	*PTPN11*			

Table S2. Mutations detected by Next Generation Sequencing (Panel of 74 genes) on the 24 baseline samples.

Patient ID	Mutation
1	KDR:NM_002253:exon11:c.1416A>T:p.Q472H 48%
1	DUSP4:NM_001394:exon4:c.983T>C :p.L328P 3.7%
1	BRAF :NM_004333:exon15:c.1799T>A:p.V600E 41%
2	BRAF:NM_004333:exon15:c.1799T>A:p.V600E 6%
2	CDKN2A:NM_001195132:exon3:c.465C>G:p.I155M 10.5%
2	CTNNB1:NM_001904:exon3:c.131_133del:p.S45del3.8%
3	NOTCH2:NM_024408:exon34:c.6956C>T:p.A2319V 47%
3	DDR2:NM_006182:exon12:c.1323G>A:p.M441I 35%
3	RAC1:NM_006908:exon2:c.85C>T:p.P29S 50%
3	MET:NM_000245:exon2:c.1124A>G:p.N375S 7%
3	BRAF :NM_004333:exon15:c.1799T>A:p.V600E 85%
3	AKT1:NM_005163:exon3:c.49G>A:p.E17K 27%
4	DDR2:NM_001014796:exon13:c.1323G>A:p.M441I 49.7%
4	BRAF:NM_004333:exon15:c.1798_1799delinsAA:p.V600K 57.8%
4	NOTCH1:NM_017617:exon33:c.6149A>G:p.N2050S 63%
4	MAP2K1:NM_002755:exon3:c.371C>T:p.P124L 37.6%
5	BRAF:NM_004333:exon15: c.1798_1799delinsAA:p.V600K 48.8%
5	IDH1:NM_005896:exon4:c.394C>T:p.R132C 19.5%
5	GRIN2A:NM_001134407:exon13:c.2714C>T:p.S905F 18.8%
5	CTNNB1:NM_001904:exon3:c.131_133del:p.S45del 2.9%
6	BRAF:NM_004333:exon15:c.1799T>A:p.V600E 26%
7	BRAF:NM_004333:exon15:c.1799T>A:p.V600E 7.3%
7	NOTCH2:NM_024408:exon34:c.6562G>A:p.A2188T 48%
8	BRAF:NM_004333:exon15:c.1799T>A:p.V600E 32,8%
8	EGFR-AS1 52.8%
9	CDKN2A NM_000077:exon2:c.457+1G>A 8%
9	BRAF:NM_004333:exon15:c.1799T>A:p.V600E 71%
10	BRAF:NM_004333:exon15:c.1799T>A:p.V600E 51%
10	BRAF:NM_004333:exon15:c.1798_1799delinsAA:p.V600K 5.2%
10	NOTCH1:NM_017617:exon33:c.6149A>G:p.N2050S 6%
10	MAP2K1:NM_002755:exon3:c.371C>T:p.P124L 4%
11	BRAF:NM_004333:exon15:c.1799T>A:p.V600E 52.9%
12	BRAF:NM_004333:exon15:c.1799T>A:p.V600E 35.5%
12	SERPINB3:NM_006919:exon4:c.284C>T:p.S95F 35.6%
13	BRAF:NM_004333:exon15:c.1799T>A:p.V600E 19.8%
13	AKT1:NM_005163:exon3:c.138C>A:p.D46E 52.3%
14	BRAF :NM_004333:exon15:c.1799T>A:p.V600E 31%
14	MET NM_001127500:exon2:c.11C>T:p.P4L 6%
15	NOTCH2:NM_024408:exon34:c.6094C>A:p.H2032N 50%
15	BRAF:NM_004333:exon15:c.1799T>A:p.V600E 58%
16	BRAF:NM_004333:exon15:c.1799T>A:p.V600E 33.5%
17	BRAF:NM_004333:exon15:c.1798_1799delinsAG:p.V600R 30%
17	FGFR3:NM_000142:exon7:c.905G>A:p.G302D 19%
18	PI3KCA:NM_006218.3:exon7:c.1173A>G:p.I391M 39%
18	BRAF :NM_004333:exon15:c.1799T>A:p.V600E 70%
19	BRAF:NM_004333:exon15:c.1799T>A:p.V600E 38%
20	BRAF:NM_004333:exon15:c.1798_1799delinsAA:p.V600K 18.6%
20	NOTCH2:NM_024408:exon34:c.7223T>A:p.L2408H 46.2%
21	BRAF:NM_004333:exon15:c.1799T>A:p.V600E 30%
22	BRAF:NM_004333:exon15:c.1799T>A:p.V600E 11%
23	BRAF:NM_004333:exon15:c.1799T>A:p.V600E 32%
24	BRAF:NM_004333:exon15:c.1799T>A:p.V600E 30%
